# Technology Trends for Massive MIMO towards 6G

**DOI:** 10.3390/s23136062

**Published:** 2023-06-30

**Authors:** Yiming Huo, Xingqin Lin, Boya Di, Hongliang Zhang, Francisco Javier Lorca Hernando, Ahmet Serdar Tan, Shahid Mumtaz, Özlem Tuğfe Demir, Kun Chen-Hu

**Affiliations:** 1 Department of Electrical and Computer Engineering, University of Victoria, Victoria, BC V8P 5C2, Canada; 2 NVIDIA, Santa Clara, CA 95050, USA; xingqinl@nvidia.com; 3 School of Electronics, Peking University, Beijing 100871, China; boya.di@pku.edu.cn (B.D.); hongliang.zhang92@gmail.com (H.Z.); 4 InterDigital Communications, Inc., London EC2A 3QR, UK; javier.lorcahernando@interdigital.com (F.J.L.H.); ahmetserdar.tan@interdigital.com (A.S.T.); 5 Instituto de Telecomunicações, 3810-193 Aveiro, Portugal; smumtaz@av.it.pt; 6 Department of Electrical and Electronics Engineering, TOBB University of Economics and Technology, Ankara 06510, Turkey; ozlemtugfedemir@etu.edu.tr; 7 Department of Signal Theory and Communications, Universidad Carlos III de Madrid, 28911 Leganés, Spain; kchen@tsc.uc3m.es

**Keywords:** 6G, massive MIMO, intelligent omni-surface (IOS), intelligent reflecting surface (IRS), cell free, artificial intelligence, vehicular communications, THz communications, non-terrestrial communications, remote sensing, inter-planetary communications

## Abstract

At the dawn of the next-generation wireless systems and networks, massive multiple-input multiple-output (MIMO) in combination with leading-edge technologies, methodologies, and architectures are poised to be a cornerstone technology. Capitalizing on its successful integration and scalability within 5G and beyond, massive MIMO has proven its merits and adaptability. Notably, a series of evolutionary advancements and revolutionary trends have begun to materialize in recent years, envisioned to redefine the landscape of future 6G wireless systems and networks. In particular, the capabilities and performance of future massive MIMO systems will be amplified through the incorporation of cutting-edge technologies, structures, and strategies. These include intelligent omni-surfaces (IOSs)/intelligent reflecting surfaces (IRSs), artificial intelligence (AI), Terahertz (THz) communications, and cell-free architectures. In addition, an array of diverse applications built on the foundation of massive MIMO will continue to proliferate and thrive. These encompass wireless localization and sensing, vehicular communications, non-terrestrial communications, remote sensing, and inter-planetary communications, among others.

## 1. Introduction

As 5G continues its rapid deployment and integration into people’s daily lives, the exploration and development of the next-generation wireless communication, commonly referred to as 6G, are poised to deliver even more remarkable performance in every domain. Table 1 provides a comparison of key performance indicators (KPIs) between 5G and 6G. Massive multiple-input multiple-output (MIMO) is a critical enabler for achieving some of the KPIs indicated in the table due to its ability to significantly increase spectral efficiency and enhance overall system performance. By utilizing a large number of antennas at the base station, massive MIMO enables spatial multiplexing and beamforming techniques, allowing for simultaneous transmission and reception of multiple data streams to multiple users. This technology improves channel capacity, reduces interference, and enhances the coverage and data rates in wireless communication systems. Massive MIMO has emerged as a pivotal technology in 5G wireless communications, witnessing unparalleled strides in development and implementation. Fueled by rapid technological advancements and significant commercial demands, massive MIMO is poised to evolve further, profoundly transforming the landscape of telecommunications and related sectors in 6G.

Intrinsic support for massive MIMO is embedded within 5G New Radio (NR) standards [3]. The initial 5G NR release, 3GPP Release 15, encompasses fundamental features facilitating massive MIMO deployment across various scenarios. Key features include reciprocity-based operation for time division duplex (TDD) systems, high-precision channel state information (CSI) feedback for multi-user MIMO (MU-MIMO), and advanced beam management for high-frequency band operation with analog beamforming. Subsequent enhancements to massive MIMO were outlined in 3GPP Release 16, including CSI feedback overhead reduction through spatial and frequency domain compression, beam management signaling overhead and latency reduction, and non-coherent joint transmission from multiple transmit and receive points (TRPs).

3GPP Release 17 heralded further evolution of massive MIMO, with angle-delay reciprocity exploited for additional CSI feedback overhead reduction. A unified transmission configuration indicator (TCI) framework was introduced to bolster multi-beam operation, accompanied by inter-cell multi-TRP enhancements and multi-TRP-specific beam management features. 3GPP Release 18 signifies the commencement of 5G Advanced, featuring further massive MIMO development. Potential areas under 3GPP investigation include uplink MIMO enhancements (e.g., the use of eight transmission antennas in the uplink and multi-panel uplink transmission), the extension of the unified TCI framework to multi-TRP scenarios, a greater number of orthogonal demodulation reference signal (DMRS) ports for MU-MIMO, and CSI reporting enhancements for user equipment (UE) with medium to high velocities.

Accelerated standardization and promising commercialization position massive MIMO as a cornerstone technology for 5G and beyond, anticipated to integrate with other enabling technologies and extend to novel verticals. This article delves into several technology trends shaping the evolution of massive MIMO en route to 6G. One such trend involves the surge of interest in intelligent surfaces [4,5], which hold significant promise for energy and cost-efficient massive MIMO. Metasurface-enabled massive MIMO can facilitate joint communications, localization, and sensing functions, thereby unlocking new use cases and bolstering wireless system performance in 6G.

The initial two sections of this article are devoted to metasurface physical fundamentals for massive MIMO and metasurface-enabled massive MIMO for localization and sensing, respectively. Following that, we offer an exploration of ultra-massive MIMO at THz frequencies, as the adoption of small wavelengths and wide bandwidth introduces unique challenges across all aspects of wireless system design. We then investigate cell-free massive MIMO technology, renowned for improving spectral and energy efficiency. Subsequently, we survey and discuss the application of artificial intelligence (AI) for massive MIMO, followed by a review of massive MIMO-OFDM for high-speed applications. As the final future-oriented application of massive MIMO leading to 6G, non-terrestrial networks (NTNs) communications has been a focal point of standardization since 2017. Therefore, we present an in-depth review, discussion, and analysis of current and future non-terrestrial applications and architectures centered around massive MIMO before drawing conclusions in this article. We have compiled a summary of the technology trends and relevant references for 6G massive MIMO in Table 2, which presents the critical advantages and challenges of each technology trend.

## 2. Metasurface-Enabled Massive MIMO

The burgeoning surge in wireless data traffic necessitates innovative communication paradigms supporting high data rates in the future 6G. Massive MIMO has attracted heated attention exploiting the implicit randomness of the wireless environment. However, traditional massive MIMO relies on the large-scale phased arrays, which induce high hardware cost and power consumption due to the energy-consuming phase shifters, especially when the number of antennas grows. This limits their scalability to support massive MIMO in practice.

Recently, intelligent metasurfaces, as a new type of ultra-thin two-dimensional metamaterial inlaid with sub-wavelength scatters, has provided a novel technology to enable or realize massive MIMO in a cost-efficient way [6]. Capable of reflecting and/or refracting the incident signals simultaneously, the surface can actively shape uncontrollable wireless environments into a desirable form via flexible phase shift reconfiguration [7]. Since such a reconfiguration of each element is usually achieved via one or two PIN diodes controlled by the biased voltage, it only involves small hardware and power costs compared to the traditional phase arrays. Consequently, the metasurface can be easily extended to a large scale, providing a practical method for realizing massive MIMO.

**Figure 1:**
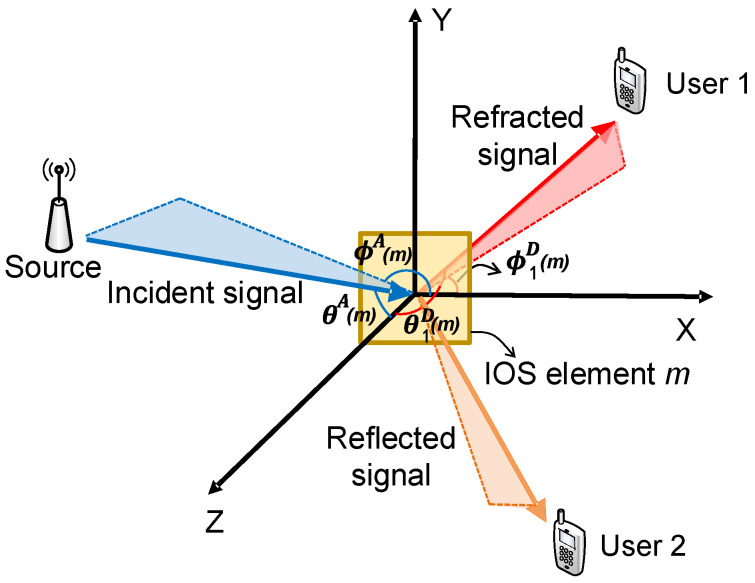
Transmission model of an intelligent surface element.

A general transmission model of one surface element is shown in Figure 1. After the incident signal arrives at the surface, part of it is reflected and the rest is refracted towards the other side. By defining the reflection–refraction ratio as ϵ, we have the reflected and refracted signals in Figure 1. Three different types of surfaces can then be classified below:

- When ϵ=0, the surface only reflects the incident signal, leading to an intelligent reflecting surface (IRS). It can be attached to the wall, serving as a reflective relay for coverage [8].
- When ϵ→∞, the surface only refracts the incident signal, serving as a reconfigurable refractive surface (RRS). It can replace the antenna array at the base station for transmission and reception [9].
- When 0<ϵ<∞, the surface can reflect and refract the incident signal simultaneously, which is defined as an intelligent omni-directional surface (IOS). Compared to IRS, it can achieve full-dimensional wireless communications despite users’ locations with respect to the surface [10].

Due to their mature manufacturing techniques and low production costs, metasurfaces have been considered as an efficient method to realize massive MIMO. The metasurface can be deployed either at the transmitter as large-aperture MIMO antennas or in the channel as passive MIMO relays. Specifically, the use of a metasurface for the realization of massive MIMO offers several notable benefits:

- Channel enhancement: The metasurface can optimize the wireless channel by reflecting or refracting the incident signals towards desired directions, thereby improving signal strength and reducing interference.
- Beamforming and steering: By controlling the phase shift across the metasurface elements, it becomes possible to create focused beams and steer them toward specific users or desired coverage areas. This enables spatially selective communications and enhances the system capacity.
- Interference management: The intelligent metasurface can actively manipulate the propagation environment to mitigate interference from nearby cells or unwanted sources, improving overall system performance.
- Energy efficiency: Compared to traditional massive MIMO systems that rely on power-hungry phase shifters, the reconfiguration of intelligent metasurfaces involves minimal hardware and power costs, making them more energy-efficient.

However, the emergence of the metasurface techniques for massive MIMO has introduced novel challenges to the field:

- As we have introduced before, the metasurface could be categorized into several types, each having its own use case [11]. It requires further investigations on what specific use cases are suitable to deploy the IRS, RRS, and IOS, respectively.
- The refracted and reflected signals of IOS are coupled with each other, determined simultaneously by the states of PIN diodes. Such a coupling effect makes it unknown whether IOS has the same impact on EM waves when the signal impinges on different sides of the surface.
- To fully exploit the refract-and-reflect characteristic of IOS, it is also necessary to explore the optimal position and orientation of the IOS given the BS and user distribution to extend the coverage on both sides of the IOS [12].
- A beamforming scheme should be reconsidered and tailored for the IOS-aided transmission since the reflected and refracted beams towards different users are dependent of each other [13].

While the aforementioned advantages and challenges focus mainly on communication aspects of the metasurface-enabled massive MIMO, it is important to note that their benefits are not restricted to communication alone. The ability to control and customize wireless environments also holds substantial promise in the area of wireless localization and sensing. As we transition towards the 6G era, these functions are becoming increasingly crucial. The following section discusses how the metasurface-enabled massive MIMO enhances wireless localization and sensing.

## 3. Localization and Sensing Using Metasurface-Enabled Massive MIMO

**Figure 2:**
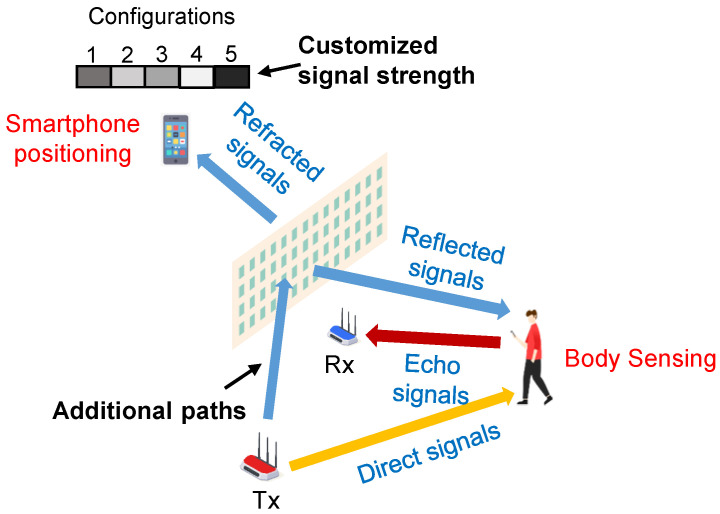
Illustration for wireless localization and sensing using metasurface-enabled massive MIMO systems.

In the forthcoming 6G era, wireless localization and sensing functionalities will be integrated into a myriad of applications, including navigation, transportation, and healthcare [14]. Consequently, there is a strong demand for services that deliver fine-resolution sensing and high localization accuracy. Massive MIMO emerges as a promising solution to this requirement, as it can narrow the beam width with a larger antenna array, resulting in high spatial resolution. However, the wireless environments in these systems are becoming complicated, for example, line-of-sight (LoS) links might be blocked by buildings or objects, degrading the accuracy of sensing and localization. Fortunately, the development of the metasurface makes it a promising solution to achieve massive MIMO, thus improving the sensing and localization accuracy by altering the propagation environment [15]. On the one hand, metasurfaces deployed in the environment can provide additional paths toward targets, extending the coverage. On the other hand, with the capability of manipulating propagation conditions, the signals from different objects or targets can be customized so that they are easier to be distinguished, as illustrated in Figure 2.

Nevertheless, the integration of the metasurface (could be IRS, RRS, or IOS according to the use case) in a wireless sensing/localization system is not trivial, which generally brings the following challenges:

- It will be a challenge to optimize the configurations relating to the metasurface [16]. Different from the designs of the metasurface for communication purposes, the optimizations here aim to maximize the sensing/localization performance, necessitating new designs. For example, the metric could be defined as the distance between two signal patterns (each corresponding to a configuration of the metasurface) from different targets/positions so that the receiver could recognize two targets/positions with less effort, leading to a higher accuracy. Moreover, as the number of metasurface elements could be large, it will cause a prohibitively high delay to enumerate all the configurations. Therefore, it will be important to select an appropriate number of configurations to achieve the trade-off between latency and accuracy.
- The coupling of the decision function with the optimization of the metasurface makes it hard to find the optimal function. To be specific, the receiver needs a decision function to transform the received signals into the information of targets/positions. As the received signals can be adjusted by the metasurface, the selection of the decision function is also influenced by the configurations of the metasurface. Therefore, a joint optimization will be necessary to improve the performance [17].
- As the spectrum is scarce in wireless systems, it is hard to spare extra bandwidth to realize the sensing function, and thus integrated sensing and communication (ISAC) becomes a natural solution [18]. Since the metasurface has different impacts on communication and sensing functions, it should be carefully configured to achieve a win–win integration of sensing and communication performance. To be specific, the mutual interference between sensing and communication can be alleviated using the metasurface. Moreover, a performance trade-off between sensing and communication needs further investigation.
- In addition to the above signal processing challenges, practical implementation is another challenge. Where to deploy the metasurface and how to determine its size should be carefully addressed, which should also take the topology of the environment into consideration.

To sum up, massive MIMO technology will be expected to provide multi-functional services integrating communication, localization, and sensing. The metasurface, which could customize the propagation environments, is believed to be an add-on enabler for future massive MIMO to facilitate such integration. In essence, the metasurface-enabled massive MIMO systems bring forth new dimensions to the wireless communication field, not only by overcoming the scalability and energy consumption issues with traditional massive MIMO, but also by extending their utility to the realms of localization and sensing. Thus, these systems are likely to play a central role in the future massive MIMO technology, serving as key enablers for multifunctional services integrating communication, localization, and sensing. However, to unlock the full potential of these systems, we must explore frequency bands that can deliver higher capacity and improved sensing capabilities. One promising area of exploration is in the realm of THz frequencies. The move towards THz frequencies will undoubtedly present new challenges that must be addressed, but it also offers exciting possibilities for the future of massive MIMO.

## 4. Ultra-Massive MIMO at THz Frequencies

Having explored the transformative potential of IOS/IRS systems for localization and sensing, we now turn our attention to the possibilities and challenges of ultra-massive MIMO at THz frequencies. The ITU-R defines THz frequencies as those in the range 0.1–10 THz, a spectrum that offers immense potential for high-capacity communications and ultra-high resolution environmental sensing. However, operating in this spectrum introduces unique challenges that demand innovative solutions, particularly in the design of transceiver architectures and network strategies.

According to ITU-R (International Telecommunication Union Radiocommunication Sector), THz frequencies are those in the range 0.1–10 THz. The lowest frequency region between 0.1 THz and 0.3 THz with the highest potential is usually called the sub-THz regime. THz and sub-THz signals serve as a bridge between radio and optical frequencies. Their wavelengths in the millimeter and sub-millimeter region make them excellent candidates to fulfill the 6G promise of extremely high-capacity communications, good situational awareness, and ultra-high resolution environmental sensing. Such small wavelengths come at the price of high uncertainty in the channel characteristics, leading to unreliable, intermittent radio links that suffer from one or several of the following impairments:

- **High path losses, molecular absorption, and blockage**: The high free-space path loss motivated by the small antenna aperture areas at these frequencies, together with the molecular absorption, blockage, diffuse scattering, and extra attenuation caused by rain, snow, or fog, lead to highly intermittent links. Link reliability must therefore be improved with the use of ultra-narrow beamforming.
- **Low energy efficiency**: RF output power degrades 20 dB per decade for a given power amplifier (PA) technology. This compromises the link budget and reinforces the need of large-scale transceivers with high numbers of antennas.
- **Large-scale transceivers**: The high beamforming gain needed to improve link reliability demands large-scale transceivers with a high number of antennas (usually, more than 1024). The sharpened, ultra-narrow beams that they produce pose significant challenges to mobility and beam tracking.
- **Phase noise**: At sub-THz/THz frequencies, CP-OFDM performance can be severely degraded by the inter-carrier interference (ICI) resulting from phase noise. Increasing the subcarrier spacing can mitigate its impact, but the correspondingly shorter symbol duration introduces a penalty in coverage and impairs the ability to mitigate large delay spreads.
- **Channel sparsity**: Ultra-narrow beams, together with ray-like wave propagation, lead to channels that exhibit small numbers of spatial degrees of freedom and ranks limited to one LoS component and a few multipath components, which challenges MIMO operation.
- **Spherical wave and near-field effects**: Large-scale transceivers exhibit significant spherical wave and near-field effects from the electrically large antenna structures that they equip, which introduces complexity to MIMO precoding strategies.
- **Beam squint**: The narrowband response of phase shifters in planar arrays introduces a frequency-dependent beam misalignment called beam squint. Losses from beam misalignments can be alleviated by using beam broadening techniques, at the cost of reduced coverage; and avoided with true time delay (TTD) units, at the cost of complexity.

There is abundant research on transceiver architectures and network solutions aimed to ameliorate some of the above issues, especially those motivated by the propagation challenges at sub-THz/THz. Among the network solutions, the aforementioned IOS/IRS/RIS equipped with very large numbers of small antenna elements are receiving considerable attention, because of their ability to tailor the characteristics of the reflected and refracted beams [4,19]. IOS/IRS/RIS at sub-THz/THz exploit ray deflections to overcome blocking and path loss; can take the benefits of the near-field effects by focusing beams to improve beamforming and 3D imaging; and can enhance the multipath richness of the channel to reinforce the spatial multiplexing capabilities at sub-THz/THz frequencies.

Among the network solutions, the aforementioned IOS/IRS/RIS equipped with very large numbers of small antenna elements are receiving considerable attention, because of their ability to tailor the characteristics of the reflected and refracted beams. This is where our discussion on cell-free massive MIMO fits in, as it is one such solution that has the potential to address these challenges.

## 5. Cell-Free Massive MIMO

After the seminal paper [20] was published, massive MIMO took place in the latest mobile 5G communications in less than ten years. Such an immediate success of a theoretical concept resulted from the foreseen performance improvement and the extreme effort put in by researchers in the last decade. Although traditional cellular massive MIMO with co-located antennas increases the data rate significantly, the user devices at the cell edges (places much far away from the serving base station) may suffer from low rates. Currently, when data communications are more dominant than voice communications in mobile systems, large data rate variations across the coverage area of the base stations are not desired. To solve the cell-edge user issues, massive MIMO scholars have developed the idea of “cell-free massive MIMO” [21], where there are no cell boundaries from the user perspective and many low-cost access points at the radio edge serve each user device [22]. Cell-free massive MIMO is envisioned as a promising technology for beyond 5G systems due to the highly improved spectral and energy efficiency it would provide. In fact, it complements the ultra-massive MIMO systems operating at THz frequencies by providing solutions to some of the aforementioned challenges. As a natural consequence, not only academia but also the industry has a great interest in cell-free massive MIMO, which is also named “distributed MIMO” or “distributed massive MIMO” by industrial researchers [23].

In a cell-free massive MIMO system, a large number of distributed low-cost access points serve multiple users’ equipment coherently using the same time-frequency resources in line with the massive MIMO operation. While densely deployed access points provide macro-diversity that increases coverage, the joint transmission/reception processing enables mitigation of the adverse effects of cell-edge interference. Indeed, cell-free massive MIMO has been developed as a clean-slate look at the coordinated multi-point processing (CoMP) in 4G. Its focus is on user-centric operation rather than network-centric to provide uniformly good service throughout the coverage.

In [21], it has been shown that cell-free massive MIMO can improve the 95%-likely per-user spectral efficiency by an order of magnitude higher than that of the traditional cellular small-cell system. In one of the following works in [24], it has been concluded that cell-free massive MIMO can also improve energy efficiency by an order of magnitude compared to cellular co-located massive MIMO. After these pioneering works were published, cell-free massive MIMO gained great importance in wireless communications. Currently, cell-free massive MIMO is treated as one of the key technologies for the next-generation mobile communication systems by the leading researchers in the area [25,26,27,28]. In [29], all the operation modes of cell-free massive MIMO have been outlined. In addition to the distributed mode that was considered in the early works, centralized cell-free massive MIMO and signal processing schemes for that are elaborated on. In the sequel, we elaborate on different aspects and the corresponding challenges for the implementation of cell-free massive MIMO in next-generation networks.

**Figure 3:**
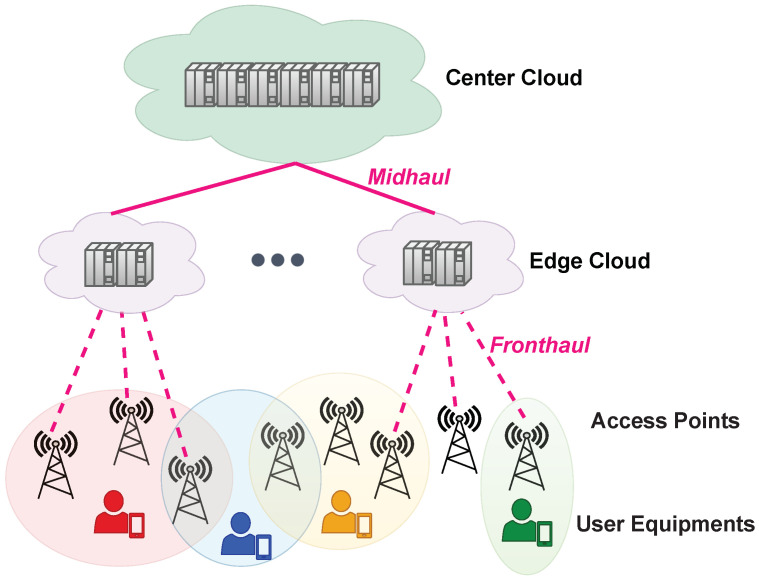
The C-RAN architecture with cell-free massive MIMO functionality.

- **Scalability:** In the original form of cell-free massive MIMO, all the access points in the network area serve all the user devices. However, such an operation of cell-free massive MIMO is not scalable in terms of signal processing complexity and fronthaul signaling load. Several network-centric and user-centric clustering methods have been developed to address the scalability issue. Although network-centric clustering can be applied in a simpler manner, user-centric clustering avoids the problem of low data rates at the cluster edges. From the CoMP literature, user-centric clustering was first considered in the cell-free context in [30,31]. Then, dividing the access points into network-centric clusters and letting each user device select a preferred subset of those clusters in a user-centric manner was proposed in [32]. In [33], a joint pilot assignment and cooperation cluster formation algorithm was proposed by analyzing the scalability of different signal processing algorithms. For a scalable (in terms of signal processing complexity and fronthaul signaling load) cell-free massive MIMO system, an access point is allowed to serve only a finite amount of user equipment [22,33]. As illustrated in Figure 3, each user equipment is served by multiple access points with the preferable channel conditions, which are the ones in the colored shaded circular regions.
- **Deployment in an end-to-end network architecture:** The physical-layer aspects of cell-free massive MIMO such as receiver combining design, transmit precoding design, and power allocation algorithms in line with a futuristic scalable system design have now been well-established [22]. In addition to the radio site aspects, the centralized computational processing unit and the fronthaul links between it and access points are two major layers in a practical cell-free massive MIMO operation envisioned to be built in 6G communication systems. When edge clouds are placed between the access points and the center cloud, as shown in Figure 3, the midhaul transport and the collaborative processing unit consisting of the edge and center cloud are the additional components in a cell-free network. Hence, the imperfections, limitations, and energy consumption should be analyzed from an end-to-end (from radio edge to the center cloud) perspective. Conducting an end-to-end study of a low-cost and energy-efficient cell-free massive MIMO implementation is critical to accelerating its practical deployment in 6G.
  The network architecture of a cell-free massive MIMO system with access points connecting to central processing units via fronthaul links is entirely in line with the wave of cloudification in mobile communications networks. Hence, it is expected from the very beginning to envision prospective cell-free networks on top of a cloud radio access network (C-RAN). In [34], the test results of a cloud-based cell-free massive MIMO implementation were reported. General-purpose processors were utilized in the central cloud. Test results demonstrate the capability of cloud-based cell-free massive MIMO to achieve 5G new radio (NR) requirements. In [35], the authors have explored the performance of cell-free massive MIMO on the virtualized C-RAN network architecture. Virtualized C-RAN enables centralizing the digital units of the access points in an edge or central cloud with virtualization and computing resource-sharing capabilities. Since digital units are located in a shared cabinet with a single cooling system, and they are orchestrated to share different computational tasks, virtualized C-RAN architecture provides several energy-saving opportunities. Going beyond virtualized C-RAN, the implementation options of cell-free massive MIMO have been discussed on top of open radio access networks (O-RAN) aiming for an intelligent, virtualized, and fully interoperable 6G architecture [36]. In [37], the end-to-end power consumption of cell-free massive MIMO in O-RAN architecture is considered together with resource allocation algorithms that jointly optimize the radio, fronthaul, and cloud processing resources in O-RAN architecture.
- **Low-cost fronthaul/midhaul transport:** Fronthaul/midhaul transport technology is one of the vital components in the low-cost deployment of cell-free massive MIMO onto the legacy network. In a large-scale cell-free massive MIMO system, deploying a dedicated optical fiber link between each access point and the edge or central cloud would be highly costly and infeasible. The so-called “radio stripes”-based fronthaul architecture developed by Ericsson reduces the cabling cost by sequentially integrating the access points into the shared fronthaul lines. When access points are distributed in a large area, other low-cost fronthaul transport technologies such as millimeter wave and terahertz wireless can both provide huge bandwidth and avoid costly wired fiber links [38]. One other option is combined fiber-wireless fronthaul/midhaul transport to balance a trade-off between link quality and cost [39]. In the latter method, the short-distance fronthaul links can be deployed wirelessly between each access point and its respective edge cloud. On the other hand, the midhaul transport from the edge to the center can benefit from extra-reliable fiber connections. Mitigating hardware impairments that naturally appear as a result of low-cost transceivers deployed at the access points and wireless fronthaul nodes is another critical aspect of the cell-free massive MIMO deployment on the legacy network.
- **Green and sustainable implementation:** In recent years, energy-saving techniques by mobile operators have gained more importance in reducing the environmental footprint and designing next-generation mobile communication systems in a green and sustainable way. Several works considered access point switching on/off methods in this research direction to save energy in a cell-free massive MIMO system. For example, in [40], the total downlink power consumption at the access points is minimized by turning off some of the access points. In addition, the virtualization and sharing of cloud and fronthaul/midhaul resources are crucial for minimizing total end-to-end energy consumption. In [35], the minimization of total downlink power consumption is considered for not only access points (radio side), but also the fronthaul and cloud computing resources. It has been shown that %14 power saving in comparison to the cellular small-cell system is possible in a small-scale system by not only benefiting from turning off access points but also turning off unused optical fronthaul resources and digital units. The results in [37] indicate that fully virtualized end-to-end resource orchestration is critical when it comes to fully benefiting from the energy-saving potential of O-RAN. At the end of the day, one should consider the limitations, energy consumption models, and the energy-saving mechanisms of digital units and processors in the edge and center cloud for the complete treatment of energy efficiency in a cell-free massive MIMO system.

In summary, one should consider the scalability aspects, the network architecture, and the respective limitations, energy consumption models, and the energy-saving mechanisms of digital units and processors in the edge and center cloud for the complete treatment of energy efficiency in a cell-free massive MIMO system. The role of AI in this context becomes critical, as it can provide smart solutions for better energy management, among other things.

## 6. Artificial Intelligence for Massive MIMO

Massive MIMO technology, empowered by artificial intelligence (AI), finds applications in Industry 5.0 [41]. This synergistic combination enables the realization of highly dependable real-time transmission of industrial 6G and other data, accompanied by reliable human–computer interaction (HCI). AI not only complements massive MIMO technology, but also offers solutions to address critical challenges encountered by ultra-massive MIMO at THz frequencies and cell-free massive MIMO systems. Massive MIMO has served as a fundamental technology in 5G networks. However, the deployment of a large number of antennas introduces new challenges, particularly the escalating cost associated with channel estimation and feedback. Additionally, there is a need for enhanced accuracy in channel estimation and prediction. The application of AI technology holds promise in addressing these issues. For example, machine learning algorithms can be applied to each categorized application area of massive MIMO, such as channel estimation [42], channel prediction [43], precoding and signal detection, and hybrid beamforming [44].

Furthermore, 6G brings new opportunities and challenges for the application of artificial intelligence (AI) models. The selection of AI models in 6G wireless communications depends on several factors, including the specific use case, available data, computational resources, and performance requirements. To guide this selection process, here are some general considerations for choosing AI models in the 6G context: The selection of AI models for wireless communications depends on various factors, including the specific use case, available data, computational resources, and performance requirements. While there is never a one-size-fits-all answer, we can provide some general considerations for choosing AI models in wireless communications:

- **Neural Networks**: Deep learning models, convolutional neural networks (CNNs) and recurrent neural networks (RNNs) have shown promising results in tasks like channel estimation, modulation classification, and signal detection. CNNs excel in extracting spatial features from signal data, while RNNs are effective for modeling temporal dependencies.
- **Reinforcement Learning**: Wireless communication systems can benefit from reinforcement learning (RL) algorithms to optimize resource allocation, power control, and spectrum management. RL models learn to make decisions based on feedback from the environment, which can lead to improved system performance.
- **Generative Adversarial Networks**: Generative adversarial networks (GANs) can be used to generate realistic synthetic data, which is valuable for data augmentation and training robust models. GANs have been applied to generate channel impulse responses, wireless signal samples, and realistic wireless channel simulations.
- **Transfer Learning**: Transfer learning allows pre-trained models from one domain to be fine-tuned for a specific wireless communication task. This approach can leverage the knowledge learned from large-scale datasets, such as image datasets or natural language processing tasks, to improve the performance of wireless communication models, especially in scenarios with limited labeled data.
- **Graph Neural Networks**: Graph neural networks (GNNs) are suitable for modeling wireless networks and their topology. GNNs can capture the spatial relationships between network nodes, enabling tasks like link prediction, network optimization, and resource allocation in wireless communication networks.

It is crucial for researchers to carefully evaluate the requirements and characteristics of their wireless communication problem and select the AI models that align with those specific requirements. Additionally, empirical validation and comparative analysis with different models can provide insights into the best-suited AI models for a given wireless communication scenario.

Nevertheless, these emerging AI technologies are not without their own set of concerns. From an industry standpoint, the primary challenges can be summarized as follows:

- It is challenging to effectively control the difference between the training data set and the actual channel. The lack of generalization of AI algorithms may lead to a decline in system performance.
- Wireless AI data and applications have their unique characteristics. However, how to organically integrate the classic AI algorithms in image and voice processing with wireless data is still unclear.
- One of the characteristics of the Massive MIMO communication system applied to Industry 5.0 is that the communication scenarios are complex and changeable (indoor, outdoor, etc.), and the business forms are diverse. Therefore, making the wireless AI solution applicable to various communication scenarios and business forms under limited computing power is a significant challenge that the industry needs to overcome.
- Developing and deploying AI models require substantial resources and energy [45]. Therefore, it is crucial to design and implement appropriate strategies to optimize the overall energy efficiency during the training and deployment processes.

On the other hand, there are several interesting trends from the research perspective. First, applying machine learning into resource allocation has the potential to achieve low complexity implementation and decrease operational costs for massive MIMO. This strategy can improve spectral efficiency and energy efficiency, increase the number of users, and decrease energy consumption as well as the time delay. Second, using machine learning or deep learning for signal detection in massive MIMO has the potential to mitigate the high complexity issues seen in the conventional linear and non-linear detection methods. Third, AI can play a potential role in interference management for massive MIMO, such as determining and predicting the number of interference sources and strengths, and further mitigating the interference. Last but not least, with massive MIMO expanding to more verticals, developing and deploying suitable segmented AI strategies for specific applications is critical. As we move forward, the integration of AI in managing and improving the performance of massive MIMO systems will become more important.

For instance, in the realm of industrial automation, specific AI strategies may be developed to optimize resource allocation, manage interference, or enhance signal detection within massive MIMO systems [41]. The authors in [46] demonstrate the utilization of deep neural networks (DNNs) to enable efficient power allocation in cell-free massive MIMO (m-MIMO) systems. The inputs for these networks are solely the large-scale channel coefficients. By assuming that these coefficients are stored locally in access points (APs), the signaling overhead is significantly reduced. The use of DNNs proves to outperform other low-complexity heuristic algorithms while maintaining acceptable performance levels. Moreover, [47] has proposed and evaluated two distinct structures of uplink (UL) DNNs. These structures enable decentralized coordinated beamforming with minimal or zero communication overhead between APs and the network controller. Both approaches rely on a priori codebook design and leverage the Received Signal Strength Indicator (RSSI) of the users. It is noteworthy that, due to advantages such as communication overhead reduction, distribute computing, adaptive learning, collaborative intelligence, federated learning can be integrated with massive MIMO. Authors in [48] have presented the integration of federated learning (FL) with a multi-user massive MIMO 6G network where they incorporate zero forcing (ZF) and minimum mean square error (MMSE) schemes at the massive MIMO base station to mitigate co-channel interference. In addition, the research work [49] presents an FL-based satellite mobile edge computing (MEC) architecture, discusses resource management and multi-modal data fusion techniques, explores data privacy and security protection using a blockchain framework. As predicted, federated learning is expected to play a crucial role in enabling future spaceborne massive MIMO communications, which will be further elaborated upon in subsequent discussions.

## 7. Massive MIMO-OFDM for High-Speed Applications

For massive MIMO, a very large number of antennas is used to either to reduce the multi-user interference (MUI), when spatially multiplexing several users, or to compensate the path loss when higher frequencies than microwave are used, such as the millimeter-waves (mm-Waves). Usually, a coherent demodulation scheme (CDS) is used in order to exploit MIMO-OFDM (orthogonal frequency-division multiplexing), where the channel estimation and the pre/post-equalization processes are complex and time-consuming operations, which require a considerable pilot overhead and increase the latency of the system. Moreover, new challenging scenarios are considered in 5G and beyond, such as high mobility scenarios (e.g., vehicular communications). The performance of the traditional CDS is even worse since reference signals cannot effectively track the fast variations of the channel with an affordable overhead.

As an alternative solution, non-coherent demodulation schemes (NCDS) based on differential modulation combined with massive MIMO-OFDM have been proposed [50]. It is shown that even in the absence of reference signals, they can significantly outperform the CDS with a reduced complexity in high-speed scenarios, where no reference signals are required. In order to successfully implement the NCDS with the MIMO-OFDM system, some relevant details should be noted as follows. First, the high number of antennas is a key aspect to successfully deploy the NCDS. In the uplink, these antennas are used as spatial combiner capable of reducing the noise and self-interference produced by the differential modulation. In the downlink, beamforming is combined with NCDS in order to increase the coverage and spatially multiplex the different users. Then, the differential modulation should be mapped in the two-dimensional time-frequency resource grid of the OFDM symbol. Different schemes are proposed: time domain, frequency domain and hybrid domain, where the latter exhibits the best performance since it can minimize required signaling to a single pilot symbol for each transmitted burst. Finally, on top of the MIMO-OFDM system, multiple users can be multiplexed in the constellation domain, which is an additional dimension to the existing spatial, time and frequency dimensions. At the transmitter, each user is choosing its own individual constellation, while at the receiver, the received joint constellation is a superposition of all individual constellations of each user. The overall performance in terms of bit error rate (BER) depends on the design of the received joint constellation, all chosen individual constellation and the mapped bits of each symbol. This non-convex optimization problem is solved using evolutionary computation, which is a subfield of artificial intelligence, capable of solving these kinds of mathematical problems.

**Figure 4:**
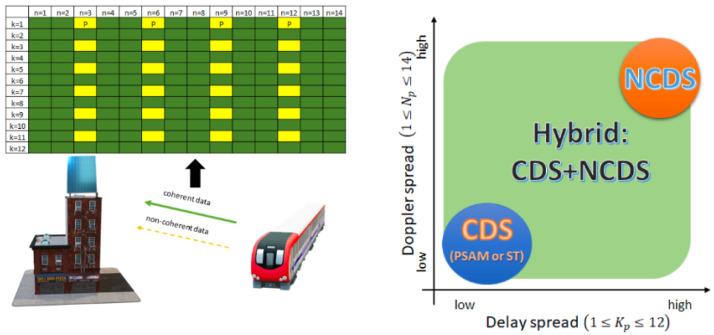
Massive MIMO for high-speed applications, a design example of hybrid demodulation scheme.

Finally, in those low- or medium-mobility scenarios, a hybrid demodulation scheme (HDS) is proposed in [51], which consists of replacing the traditional reference signals in CDS by a new differentially encoded data stream that can be non-coherently detected. The latter can be demodulated without the knowledge of the channel state information and subsequently used for the channel estimation. A design example is illustrated in Figure 4. Consequently, HDS can exploit both the benefits of a CDS and NCDS to increase the spectral efficiency. It is outlined that the channel estimation is almost as good as CDS, while the BER performance and throughput are improved for different channel conditions with a very small complexity increase.

These advancements in massive MIMO-OFDM for high-speed applications on the ground may also provide a strong foundation for the adaptation of the technology for non-terrestrial communications, which brings a new set of challenges and opportunities as detailed in the following section.

## 8. Massive MIMO for Non-Terrestrial and Deep-Space Communications

With the completion of 5G standardization phases 1 and 2, as defined by 3GPP release 15 and 16, the first half of 2022 brought about the third evolution of the global 5G standard, culminating in the system design completion through 3GPP release 17. This release is recognized as a significant expansion of 5G, accommodating a host of new devices and applications. Notably, 3GPP release 17 has brought 5G NR support to satellite communications, a key constituent of non-terrestrial networks. Essentially, the NTN concept includes any network incorporating aerial or space-based elements, with satellite communication networks, high-altitude platform systems (HAPS)—including aircraft, balloons, and airships—and air-to-ground networks forming the NTN family [52].

**Figure 5:**
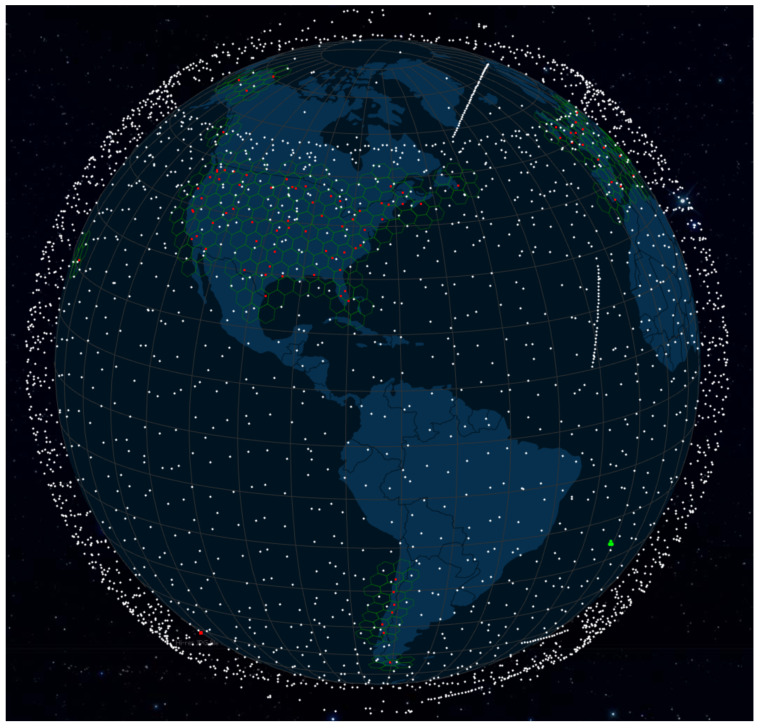
Illustrations of real-time orbital and location information of satellites in SpaceX Starlink constellation (as of 16 May 2023).

The primary objective of 3GPP NTN is to enable advanced features like ubiquitous connectivity and coverage for remote and rural regions through satellite communication networks. These networks serve two distinct functions: facilitating satellite backhaul communications for application scenarios such as customer–premises equipment (CPE) and direct low data rate services for handhelds, and adapting eMTC (enhanced Machine Type Communication) and NB-IoT (Narrowband Internet-of-Things) operation for satellite communications. Recent years have seen a surge of interest and development in Low Earth Orbit (LEO) satellites, which enable broadband access and services [54]. Among the significant players in this field, including Starlink (SpaceX), Kuiper (Amazon), OneWeb, Boeing, and Telestat, Starlink is the frontrunner, owing to the scale and dimension of its satellite megaconstellation and its service subscriber base. As illustrated in Figure 5, a total of 4288 Starlink satellites have been launched, with 3526 of them currently in active service as of 16 May 2023 [53].

Several key factors have spurred the rapid growth of spaceborne broadband access [54], such as a decrease in launch costs, private investment, the widespread implementation of AI and cloud/edge computing, and advances in satellite wireless and networking technologies. From the perspective of wireless communications and particularly massive MIMO, an overview of trends, challenges and possible solutions is provided below.

- **Competition and coexistence**: The deployment and operation of expanding satellite constellations below 2000 km present challenges in managing competition and facilitating coexistence. Many LEO megaconstellation developers aim to deploy satellites as low as possible to minimize propagation delay, while the ITU follows a “First Come, First Served” approach in accessing orbit/spectrum resources through the ITU cooperative system. With increasing congestion and reported collision incidents in LEO [54], regulatory measures, strategies, and technologies are needed to manage growing space traffic and ensure safe disposal of satellites/spacecraft. With the escape velocity being at 7.8 km/s, tracking, localizing the satellites/spacecraft and further enabling collision-avoidance can be difficult even with contemporary AI-assisted sensing and detecting technologies.
- **Spectrum management**: Furthermore, spectrum management is a pivotal factor as the scarcity of spectrum resources introduces significant hurdles. To enable 5G NR for NTN, 3GPP release 17 has embarked on investigations to support satellite backhaul communication for CPEs and facilitate direct links to handheld devices for low data rate services using the sub-7 GHz S-band. Frequencies beyond 10 GHz will be the subject of research in 3GPP release 18. Concurrently, Starlink’s first-generation system, Gen1, has predominantly utilized Ku-band and Ka-band for diverse link types and transmission directions, with the second-generation (Gen2) intending to incorporate the V-band. The use of either sub-7 GHz S-band or Ku/Ka/V-band could, to a certain extent, overlap with the spectrum of ongoing 5G and upcoming 6G systems, along with other systems operating within these bands. This overlap could instigate interference and co-existence issues among various systems and networks. Both SpaceX and OneWeb have voiced concerns over potential interference encountered by non-geostationary orbit (NGSO) satellite internet if terrestrial 5G employs the 12 GHz band. The push to support more satellite direct links to user equipment (UE) using the sub-7 GHz S-band could further amplify these interference challenges. Thus, an in-depth exploration of spectral resources (e.g., higher frequency bands) and spectrum management for spaceborne massive MIMO is anticipated.
- **Interference Management**: Moreover, a range of interferences can arise within a single space network and between different space networks [55]. For instance, in-band/out-band interference (or emission) can occur between user terminals (UTs) and ground stations within the same megaconstellation. In scenarios involving multiple space networks, satellite transmissions from one megaconstellation could interfere with the reception by UTs and ground stations of other megaconstellations. Similarly, transmissions from UTs/ground stations could cause interference with satellites in different constellations. Traditionally, co-existing space networks mitigate interference through the shared coordination of frequency allocations (both uplink and downlink). Nevertheless, the advent of increasingly complex scenarios necessitates the development of more sophisticated interference mitigation technologies.
- **Channel modeling**: Additionally, various propagation conditions, as outlined by the ITU, can introduce interference to the satellite system. These conditions include line of sight, diffraction, tropospheric scatter, surface ducting, elevated layer reflection/refraction, and hydrometeor scatter. Consequently, the construction of precise channel models for massive MIMO satellite communications must take these physical conditions into account. The severe Doppler effect, induced by the high mobility of satellites and UTs, along with the longer propagation latency, complicates the acquisition of instantaneous channel state information (iCSI) at the transmitter [56]. As a result, spaceborne multi-user massive MIMO systems must strive to develop more cost-effective algorithms for computing precoding vectors.
- **Hardware availability and readiness**: From a hardware standpoint, the RF performance of antennas and transceivers in multi-beam satellites is paramount as it influences key factors such as the EIRP (effective isotropic radiation power), frequency reuse, inter-beam interference, co-channel interference, adjacent channel interference, beam management, and more. At the satellite/ground station end, high-gain antennas like multi-beam reflector antennas and phased-array antennas are commonly utilized. For user terminals (UTs), the trend is towards adopting high-frequency (e.g., mmWave) phased-array antennas for high-throughput applications. The catalyst stems from the constant improvement of the antenna and integrated circuit designs, which makes high-efficiency RF front ends more available and affordable [57].
- **Artificial intelligence and cloud/edge computing**: In addition, certain emerging technologies have emerged as vital accelerators for the deployment of space broadband. In comparison to twenty years ago, AI and cloud/edge computing technologies have become instrumental in enabling high-performance satellite broadband connectivity and services. These services include beam-hopping, interference management, satellite traffic control, image processing, mapping, and computation offloading [58].
  In May 2021, Google clinched a deal to provide Starlink with networking resources, utilizing its private fiber-optic network for swift cloud connections. Instead of outsourcing, SpaceX will incorporate ground stations in Google’s data centers for secure, intelligent connectivity. Likewise, in 2020, Microsoft joined forces with SpaceX to link Starlink with its global network, including Azure edge devices, aiming to facilitate satellite connectivity for field-deployed assets worldwide. Amazon Web Services, offering sophisticated cloud computing platforms, is positioned to integrate with Amazon’s LEO satellite constellation, Project Kuiper.
  These technological developments underpin the critical role of massive MIMO in future satellite communications. Massive MIMO systems can further improve the efficiency and performance of these AI-powered and cloud-integrated satellite networks by offering superior capacity, link reliability, beam and interference management, and spectrum efficiency improvement. The combination of these advanced technologies will shape the future of spaceborne broadband connectivity, thereby further promoting a more prosperous space era. Thus, it is anticipated that we will see a rise in the number of cloud/edge computing and AI-empowered satellite constellations in 2023 and beyond, ultimately emphasizing the profound importance and potential of massive MIMO in this domain.

The insights and discourse presented thus far are rooted in communication scenarios that exist within the confines of the Near-Earth Space (NES). The NES, by definition, encompasses the region stretching from the neutral terrestrial atmospheric layers (160–200 km) up to the lunar orbit, approximately 384,400 km away [59]. However, the potential application areas of massive MIMO communication are not strictly limited to these parameters and can be extended to encompass broader and more distant domains presented as follows.

**Figure 6:**
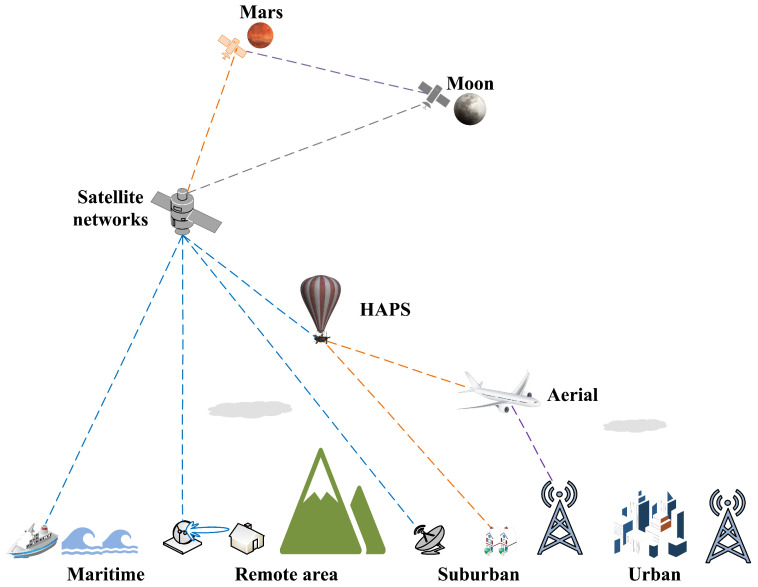
Illustrations of massive MIMO for non-terrestrial and deep-space networks.

**Figure 7:**
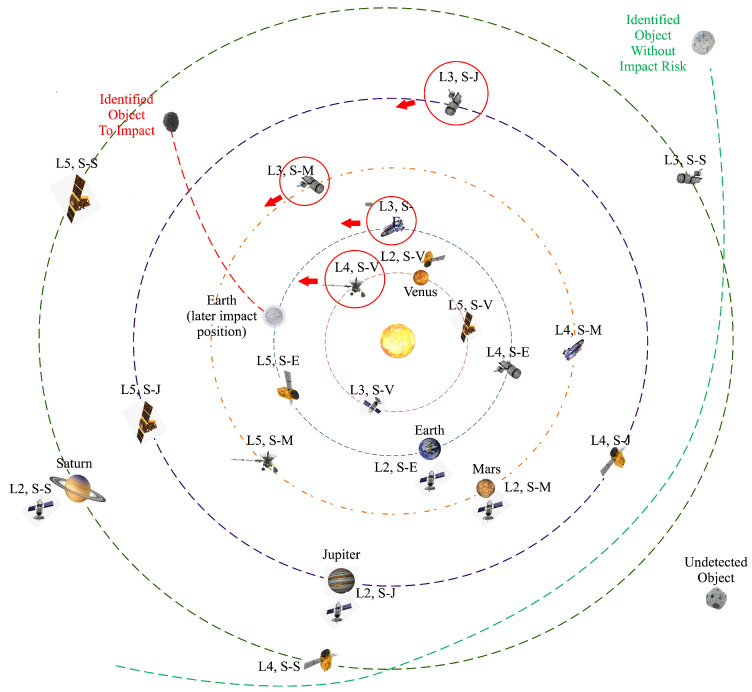
Illustrations of the SCADN framework which monitors the space, and detects and intercepts the hazardous space objects (dimension of celestial bodies, space objects, and orbits are not scaled) [63].

- **Broader NTN Networks**: From a more encompassing standpoint, there is a growing trend where NGSO megaconstellations are increasingly supplementing or effectively co-operating with geosynchronous Earth orbit (GEO) networks, high altitude platform systems, air-to-ground networks, drone networks, maritime networks and even beyond as illustrated in Figure 6. The actualization of such an expansive NTN ecosystem may pose significant challenges, including managing the co-existence and competition between networks and fostering the development of massive MIMO technologies tailored to various types of applications. In contrast, the reach of space-enabled networks and massive MIMO is expected to extend beyond the near-Earth space. To illustrate, NOKIA Bell Labs is slated to construct and launch the first compact, low-power, space-durable, end-to-end LTE network on the Moon as part of NASA’s Artemis program, which aims to return astronauts to the Moon by 2024 [60,61]. This groundbreaking endeavor establishes the vital communication bedrock that future massive MIMO technology must evolve to meet the demands of prospective Earth–Moon communications.
- **Massive MIMO in Deep Space**: Anticipating humanity’s transition to a multiplanetary existence [62], the deployment of telecommunication infrastructure and massive MIMO technologies becomes essential in accommodating more distant celestial bodies. As proposed in the Solar Communication and Defense Networks (SCADN) concept [63], a comprehensive massive MIMO sensing and communications system, rooted in an interconnected network of countless spacecraft and satellites strewn across the solar system, presents a promising solution as illustrated in Figure 7. This integrated framework offers capabilities for early threat identification and neutralization (for instance, hazardous asteroids or comets), thus safeguarding both our home planet and potential extraterrestrial human outposts. Furthermore, it lays the groundwork for wireless connectivity across the solar system, a critical infrastructure as humans venture into establishing colonies on other celestial bodies. However, this ambitious objective is not without significant challenges. The colossal distances and consequent communication delays between Earth and other celestial bodies demand innovative and robust solutions. Leveraging breakthroughs in artificial intelligence, machine/deep learning, edge computing, edge AI, and distributed and federated learning could offer the necessary tools to overcome these hurdles and realize this vision.

To sum up, the massive MIMO technology has been fast extending the communicating and sensing capabilities of humanity beyond the terrain and even Earth, which will undoubtedly facilitate a more prosperous space era for all mankind.

## 9. Conclusions

This article undertakes an in-depth exploration of the technological evolutions underpinning the future of massive MIMO, a critical element as we transition into the 6G era. We begin with a detailed overview of the contemporary advancements in massive MIMO standardization and research, drawing attention to the intricate dynamics shaping this arena. Further, we delve into the potential of IRS/IOS technologies to revolutionize massive MIMO communications and the associated challenges, particularly in harnessing IRS/IOS to bolster localization and sensing capabilities.

We continue our exploration with an inspection of ultra-massive MIMO at THz frequencies, uncovering the complex considerations influencing its design. The spotlight then turns to cell-free massive MIMO architecture, a promising innovation with the capacity to dramatically enhance the spectral and energy efficiencies of wireless systems and networks. In parallel, we outline the hurdles and emergent trends in the application of AI to massive MIMO, emphasizing its increasing relevance in future vertical applications.

Additionally, we offer a practical analysis of massive MIMO-OFDM-enabled high-speed communications, featuring tangible design examples to illustrate their potential. The review concludes with a rigorous investigation into the evolving landscape of massive MIMO communications in non-terrestrial networks, where the emphasis lies on the frontier spaces of near-Earth and deep space.

This conclusion highlights the importance of further exploring these technologies, given their potential to expand and transform our communication capabilities beyond terrestrial confines. We underscore the significance of envisioning and preparing for this transformative shift in communication paradigms, from Earth-bound networks to expansive non-terrestrial systems such as in other celestial bodies across the solar system. In the final analysis, these technologies promise to deliver unprecedented levels of connectivity, redefining our understanding of what is possible within the realm of communication technology.

## Figures and Tables

**Table 1:** Comparison of 5G and 6G KPIs [1,2].

KPI	5G	6G
Peak Data Rate	20 Gb/s	≥1 Tb/s
Experienced Data Rate	0.1 Gb/s	1 Gb/s
Peak spectral efficiency	30 b/s/Hz	60 b/s/Hz
Experienced spectral efficiency	0.3 b/s/Hz	3 b/s/Hz
Operating bandwidth	400 MHz for sub-6 GHz 3.25 GHz for mmWave	400 MHz for sub-6 GHz 3.25 GHz for mmWave 10–100 GHz for THz bands
Carrier bandwidth	400 MHz	To be specified
Latency	1 ms	100 ms
Connection Density	$10^{6}$ devices/km^2^	$10^{7}$ devices/km^2^
Area traffic capacity	10 Mb/s/m^2^	1 Gb/s/m^2^
Mobility	500 km/h	1000 km/h
Energy Efficiency	not specified	1 Tb/J
Jitter	not specified	1 μs
Reliability	500 km/h	1000 km/h

**Table 2:** An overview of technology trends and related references in 6G massive MIMO.

Technology Trends	Advantage	Challenge	Ref.
Metasurface- enabled Massive MIMO	Channel enhancement, Beamforming and steering, Interference alleviation, Energy-efficient communication	Type selection, Coupling effect, Deployment, Beamforming scheme	[6,7,8,9,10,11,12,13]
Localization & Sensing	Customize channels, Enlarge differences	Configuration, Joint optimization, Communication integration, Practical implementation	[14,15,16,17,18]
Ultra- Massive MIMO at THz Frequencies	High capacity, Ultra-high resolution environmental sensing, Tailoring beam characteristics	Intermittent and unreliable links, Large transceivers and mobility, Phase noise, Channel sparsity, Spherical wave and near- field effects, Beam squint	[19]
Cell-Free Massive MIMO	Improved spectral and energy efficiency, Uniform service provision, Cloudification and virtualization	Hardware impairments Cost-effective fronthaul /midhaul transport	[20,21,22,23,24,25,26,27,28,29,30,31,32,33,34,35,36,37,38,39,40]
Artificial Intelligence	Reliable real-time transmission, Improved channel estimation and prediction	Integration of AI with wireless data, Segmented AI for specific applications	[41,42,43,44,45,46,47,48,49]
High-speed Applications	Non-coherent demodulation, Multi-user multiplexing	High mobility Non-convex optimization	[50,51]
Non-terrestrial & Deep-space	Ubiquitous connectivity & sensing via satellites & deep space Multi-planetary prosperity	Enormous propagation loss & delay, Distributed & edge computing	[52,53,54,55,56,57,58,59,60,61,62,63]

## Data Availability

Not applicable.
